# Risk factors of white matter hyperintensities in migraine patients

**DOI:** 10.1186/s12883-022-02680-8

**Published:** 2022-04-29

**Authors:** Jasem Yousef Al-Hashel, Raed Alroughani, Khaled Gad, Lamiaa Al-Sarraf, Samar Farouk Ahmed

**Affiliations:** 1 Department of Neurology, Ibn Sina Hospital, P.O. Box 25427, Safat, 13115 Kuwait City, Kuwait; 2 Department of Medicine, Faculty of Medicine, Health Sciences Centre, Kuwait University, P.O. Box 24923, Safat, 13110 Kuwait City, Kuwait; 3 Division of Neurology, Department of Medicine, Amiri Hospital, Sharq, Kuwait; 4 Medical imaging Department, Ibn Sina Hospital, P.O. Box 25427, 13115 Safat, Kuwait; 5 Radiology Department, Suez Canal University, Ismailia, Egypt; 6 Neuropsychiatry Department, Faculty of Medicine, Al-Minia University, P.O. Box 61519, Minia City, Minia 61111 Egypt

**Keywords:** Migraine with aura, Migraine without aura, Episodic migraine, Chronic migraine, Magnetic resonance imaging, Homocysteine

## Abstract

**Background:**

Migraine frequently is associated with White Matter Hyperintensities (WMHs). We aimed to assess the frequency of WMHs in migraine and to assess their risk factors.

**Methods:**

This is cross-sectional study included 60 migraine patients of both genders, aged between 18 and 55 years. Patients with vascular risk factors were excluded. We also included a matched healthy control group with no migraine. Demographic, clinical data, and serum level of homocysteine were recorded. All subjects underwent brain MRI (3 Tesla).

**Results:**

The mean age was 38.65 years and most of our cohort were female (83.3). A total of 24 migraine patients (40%) had WMHs versus (10%) in the control group, (*P* < 0.013). Patients with WMHs were significantly older (43.50 + 8.71 versus. 35.92+ 8.55 years, *P* < 0.001), have a longer disease duration (14.54+ 7.76versus 8.58+ 6.89 years, *P* < 0.002), higher monthly migraine attacks (9.27+ 4. 31 versus 7.78 + 2.41 *P* < 0.020) and high serum homocysteine level (11.05+ 5.63 versus 6.36 + 6.27, *P* < 0.006) compared to those without WMHs. WMHs were more frequent in chronic migraine compared to episodic migraine (75% versus 34.6%; *P* < 0.030) and migraine with aura compared to those without aura (38.3% versus 29,2; *P* < 0.001). WMHs were mostly situated in the frontal lobes (83.4%), both hemispheres (70.8%), and mainly subcortically (83.3%).

**Conclusion:**

Older age, longer disease duration, frequent attacks, and high serum homocysteine level are main the risk factors for WMHs in this cohort. The severity or duration of migraine attacks did not increase the frequency of WMHs. The number of WMHs was significantly higher in chronic compared to episodic migraineurs.

## Introduction

Migraine is characterized by recurrent moderate to severe headache attacks, sometimes associated with autonomic symptoms [1]. Migraine has a high prevalence and a high social-economic burden that affects the quality of life. Globally, migraine affects approximately 15% of the general population [2]. Our national survey showed that the prevalence of migraine was 24% of the Kuwaiti population [3].

Recent imaging technology provided more convenient methods for better understanding the pathological mechanism of migraine and revealing abnormal brain regions associated with migraine. Previous MRI studies reported functional and structural abnormality in migraine patients and have suggested that brain malfunctioning may be associated with migraine pathophysiology [4–6]. On the other hand, recurrent and long-term migraine attacks may lead to functional and structural changes that may underlie the progression of the disorder [7, 8].

Migraine sometimes is associated with White Matter Hyperintensities (WMHs) [9], which are hyperintense brain lesions in T2-weighted and Fluid-Attenuated Inversion Recovery (FLAIR) images [10]. The relation between WMHs and the clinical features of migraine is still unclear. Some studies suggested that WMHs are more frequent in migraine patients with more headache attacks [9]. Other studies demonstrated that disease duration was associated with WMHs in migraine patients [11]. One study found an association between WMHs and the older age of patients [12]. Other studies have reported that WMHs are not associated with disease duration or attack frequency [13, 14]. Understanding the relation between WMHs and migraine may help patient management.

Previous population-based studies have indicated that WMHs in migraine are not associated with stroke or the decline in cognitive function [15, 16]. Eggers proposed that WMHs might be caused by multiple micro emboli, which are induced by platelet aggregation abnormalities usually observed in migraine patients [17]. This supports that WMHs may reflect an abnormal internal environment in patients.

A previous study has shown that hyperhomocysteinemia is associated with migraine, especially migraine with aura [18]. A high level of homocysteine is associated with vascular insults such as thrombosis, atherosclerosis, and ischemic diseases, such as myocardial infarction and ischemic stroke [19–21]. Hyperhomocysteinemia may stimulate the initiation of migraine attacks through changes in pain threshold [22]. A previous study revealed that higher levels of homocysteine are related to higher severity and frequency of migraine attacks [23], whereas others showed no relationship between homocysteine levels and frequency of these attacks [24]. Association between elevated serum homocysteine level and WMHs have been documented in several studies [25–27].

This study aimed to assess the frequency and location of WMHs in migraine patients and to assess its relation with headache duration, frequency, severity of migraine attacks, and the serum level of homocysteine.

## Method

Eighty subjects were included in this cross-sectional study, comprising 60 migraine patients and 20 normal control subjects (NCs). Migraine patients were recruited from the outpatient headache clinic of the department of neurology, Ibn Sine Hospital, Kuwait. The patients were diagnosed with migraine based on the International Classification of Headache Disorders (ICHD-III) [1]. Episodic migraine (EM) is defined as migraine attack days being less than 15 days per month. Chronic migraine (CM) is defined as headache attack days being more than 15 days per month. The definition of migraine refers to 1.1 migraines without aura, a 1.2 migraine with aura, and 1.3 refers to CM in the International Classification of Headache Disorders, Third Edition (ICHD-III) [1]. Inclusion criteria were age 18–60 years old, both genders, willing to participate in the study. NCs were voluntarily recruited from clinical staff and were matched with migraine patients in terms of gender, age, educational years, and hand predominance. Normal controls should not have any metabolic or neurologic diseases. Also, they have never experienced any other type of headache and have no family members who suffered from a migraine.

Subjects were excluded from the study if they have medical or psychiatric disorders such as hypertension, diabetes mellitus, hypercholesterolemia, B12 deficiency, cardiovascular diseases, connective tissue diseases, neoplasm, infection, stroke, head trauma, other subtypes of headache, any other neurological disorder, smoking, women taking oral contraceptives, chronic somatic pain, severe anxiety or depression, BMI greater than 30, substance abuse or contraindications for MRI. Inclusion and exclusion criteria of NC were similar to those of the patients. All neurological and psychiatric diseases were excluded based on clinical examination structured interviews and investigations. All subjects were screened and examined by a neurologist to ensure that they met the inclusion/exclusion criteria.

Demographic and clinical data, including age, gender, disease duration, attack frequency (times/month), attack duration (hours), and their scores on the visual analog scale (VAS) [28], the Migraine Disability Assessment Scale (MIDAS) [29], the Headache Impact Test (HIT-6) [30] and the Montreal Cognitive Assessment (MoCA) [31] were recorded. Ten-point VAS to assess the severity of pain 0 means no pain and 10 means severe pain. MIDAS score is derived from five questions about missed time from work, household work, and missed days of nonwork activities. and days at work and days of household activities where productivity was reduced (social, family, and leisure activities) due to migraine in the last 3 months. The MIDAS score was the sum of missed workdays, household chores days, non-work activity days, days at work, and days of household activities where productivity was reduced. The HIT-6 items assess the impact of headache on social functioning, role functioning, vitality, cognitive functioning, and psychological distress. The HIT-6 also measures the severity of headache pain.

The higher the MIDAS score, the more severe the disability caused by migraine. Both patients and NC underwent Montreal Cognitive Assessment (MoCA). The total score of MoCA is 30 and when the score falls below 26, cognitive impairment is present. The lower the MoCA score is, the worse the cognitive function.

Fasting Blood samples were collected to measure the serum homocysteine level. Hyperhomocysteinemia was defined as serum levels higher than 15 μmol/l) (reference range 5.00–15.00 lmol/l).

All Magnetic Resonance (MRI) studies were acquired using a 3.0 Tesla Siemens Trio Tim MRI scanner with a 12-channel head coil. Subjects with obvious structural abnormalities and lesions in the brain were excluded from the study.

MRI studies acquisition was ongoing in the interictal stage. The imaging protocol included the following anatomical sequences; 3D T1 mpr echo (*T1-weighted image/multiplanar reconstruction*) in the sagittal view to obtain isotropic anatomical data; 3D FLAIR (*fluid-attenuated inversion recovery*) in the sagittal view with an isotropic voxel; DWI (*diffusion-weighted image*) in the axial view; SWI (*susceptibility-weighted imaging*) in the axial and coronal view. A minimum of 3-mm size threshold was applied to define a hyperintensity. The 4 mm slice thickness is the one we typically use in our multiplanar reconstruction algorithm however, an isotropic 0.8 mm slice thickness was used in our volumetric (pre-reconstruction) source images resulting in 95% slice resolution. The total lesion count was manually calculated. Radiologists blind to clinical data assessed the brain image. WMHs were defined according to the location: lobes (frontal, parietal, temporal, occipital), hemisphere (one and both hemispheres). Since there has been no solid classification criteria in the literature of T2 hyperintensities in migraine patients, we assumed that categorizing the lesions into different compartments rather than simply dichotomizing them into supratentorial or infratentorial would have be more informative and may end up with new findings. Moreover, it’s been reported that hyperintensities matching both Barkhof and McDonalds criteria could be present in Headache. Lesions were subclassified as cortical/juxtacortical, subcortical, periventricular, infratentorial according to the Barkhof and McDonalds criteria. Deep/periventricular lesions are those not abutting the inner layer of the cortex on any of the 3 orthogonal planes by 3-T MRI scanning [32, 33].

Then, two independent researchers, a radiologist and a neurologist experienced in neuroimaging assessed the presence and anatomical location of WMHs. In cases of doubt, a decision was made by consensus.

Our study was in accordance with the 1964 Helsinki declaration and its later amendments or comparable ethical standards [34]. Written informed consent was obtained from all migraine patients and normal controls. This study was partly funded by a scientific grant from Kuwait University, Research Sector Faculty of Medicine (MM01/19).

### Data analysis

Data obtained from migraine patients were analyzed to describe the imaging characteristics of WMHs. Furthermore, the association between WMHs and the clinical features of migraine and serum Homocysteine was investigated. Statistical analysis was performed with IBM SPSS 25.0 (IBM Corp., Armonk, NY, USA). Demographic and clinical characteristics were reported using descriptive statistics including percentages and means. The Chi-square test was used for the comparison of categorical data. The student’s t-test was used to compare the means of normally distributed variables. Pearson correlation analysis was used for the data with normal distribution. Spearman correlation analysis was used for the data without normal distribution. A value of *P* < 0.05 was considered statistically significant.

## Results

### Demographic and clinical data

Table 1 displays the demographic and clinical data of migraine patients and the control group. Age and gender did not significantly differ between the migraine patients and controls.

**Table 1:** Clinical and demographic character of migraine patients (Number = 60) and control group (Number = 20)

Variables	Migraine Cohort	NC Group	P
	Mean **+** SD/Number (%) ***N*** = 60	Mean **+** SD/Number (%) ***N*** = 20	
**Mean Age**	38.65 + 8.45	35.80+ 8.20	0.633
**Range**	23–55	21–55	
**Gender**			
• Male	• 10 (16.7)	• 3 (15)	0.861
• Female	• 50 (83.3)	• 17 (85)	
**Mean MOCA score**	28.90 + 0.93	29.00 + 1.03	0.834
**Range**	26–30	26–30	
**Frequency of** WMH **in MRI**	24 (40.0)	2 (10)	0.013*
**Mean Serum level of Homocysteine**	7.80+ 4.48	6.01 + 1.11	0.016*
**Range**	4.10–18.50	4.10–7.80	

*NC* normal control, *SD* standard deviation, *WMHs* White matter hyperintensities

*A significance level, *P* < 0.05

Twenty-four migraine patients (40%) had WMHs versus 2 (10%) in the NC group, (*P* < 0.013). Migraine patients have a similar MOCA score compared to the control group (28.90 + 0.93 versus 29.00 + 1.03, *P* < 0.834). Despite the mean serum homocysteine level among patients with WMHs being within the normal reference range, it was significantly higher in the migraine group compared to the control group (7.80+ 4.48 versus 6.01 + 1.11 *P* < 0.016).

Table 2 showed the clinical character and impact of migraine in our cohort. 29 (48.3%) used prophylactic treatment and all of our cohort used symptomatic treatment. Topiramate, beta-blockers, a tricyclic antidepressant, and onabotulinumtoxin A were reported as preventive medications. Triptans, non-steroidal anti-inflammatory and paracetamol were used as pain killer in our cohort. The mean numbers of analgesic tablets were 18.32 ± 10.46 and the mean numbers of analgesic days were 12.11 ± 6.69.

**Table 2:** Clinical character and impact of migraine (Number = 60)

Variables	Migraine Cohort
	Mean **+** SD/Number (%) ***N*** = 60
**Diagnosis**	
• Episodic Migraine	• 52 (86.7)
• Chronic Migraine	• 8 (13.3)
• Migraine with aura	• 12 (20)
• Migraine without aura	• 48 (80)
**Mean disease duration in years**	10.97+ 7.56
**Range**	1–29
**Mean Frequency of migraine headache/ month**	5.65+ 6.36
	1–30
**Mean duration of migraine headache in hours**	14.19+ 12.02
**Range**	2–72
**Mean number of analgesic days**	12.11 ± 6.69.
**Use of prophylactic treatment**	29 (48.3)
**VAS**	8.30+ 1.67
**Range**	2–10
**Mean HIT 6 score**	64.12 + 10.29
**Range**	39–78
**Mean MIDAS score**	51.42 + 57.70
**Range**	0–240

*HIT-6* Headache Impact Test, *MIDAS* Migraine Disability Assessment Scale, *MoCA* Montreal Cognitive Assessment, *NA* not available, *NC* normal control, *SD* standard deviation, *VAS* visual analogue scale, *WMHs* White matter hyperintensities

*A significance level, *P* < 0.05

### Relations of WMHs to migraine characters

Table 3 showed that patients with WMHs were significantly older (43.50 + 8.71 versus. 35.92+ 7.02 years, *P* < 0.001). WMHs were prevalent in both genders. They are significantly frequent in chronic migraine compared to episodic migraine and in migraine with aura compared to those without aura. When examining the association between the disease duration and the presence of WMH, it was found that migraine patients with WMHs have a longer disease duration than patients without WMH (14.54+ 7.76versus. 8.58+ 6.49 years, *P* < 0.002). Migraine patients with WMH have higher monthly migraine attacks compared to those without WMHs (7.96+ 5.53 versus 4.11 + 6.48, *P* < 0.020). There is no significant relationship between the duration of headache attacks and the severity of headache pain with the occurrence of WMHs. Despite the mean serum homocysteine level among patients with WMHs being within the normal reference range, there was a significant relationship between high serum homocysteine level and the occurrence of WMH (9.27+ 4.31 μmol/L versus 7.78 + 2.41 μmol/L, *P* < 0.006). Significant migraine disability measured with HIT-6 and MIDAS scores was significantly associated with WMHs. The cognitive assessment measured with MoCA scores was not associated with a significant risk of WMHs in migraine patients.

**Table 3:** Comparison of clinical characteristics between the non-WMH group and WMH group

Variable	Non-WMHs group (***n*** = 36)	WMHs group (***n*** = 24)	***P*** value
**Age**	35.92+ 7.02	43.50 + 8.71	0.001*
**Gender**			
• Male	• 5 (50)	• 5 (50)	0.480
• Female	•31 (62)	•19 (38)	
**Mean Disease Duration**	8.58+ 6.49	14.54+ 7.76	0.002*
**Mean Frequency of headache/month**	4.11 + 6.48	7.96+ 5.53	0.020*
**Mean duration of headache attach in hour**	15.25+ 10.29	12.17+ 14.20	0.333
• Episodic Migraine	• 34 (65.4)	• 18 (34.6)	0.030*
• Chronic Migraine	• 2 (25)	• 6 (75)	
• Migraine with aura	• 2 (16.7)	• 10 (83.3)	0.001*
• Migraine without aura	• 34 (70.8)	• 14 (29.2)	
**VAS**	8.28+ 1.83	8.33 + 1.43	0.901
**Mean HIT 6 score**	60.81+ 9.64	69.25 + 9.02	0.001*
**Mean MIDAS score**	41.33+ 7.90	74.04 + 13.61	0.030*
**Mean MOCA score**	28.89 + 1.01	28.91+ 0.83	0.659
**Mean Serum level of Homocysteine**	7.78+ 2.41	9.27 + 4.31	0.006*

*HIT-6* Headache Impact Test, *MIDAS* Migraine Disability Assessment Scale, *MoCA* Montreal Cognitive Assessment, *NA* not available, *NC* normal control, *SD* standard deviation, *VAS* visual analogue scale, *WMHs* White matter hyperintensities

*A significance level, *P* < 0.05

We did not address significant correlation between occurrence of WMHs and the use of prophylactic medication (r = 0.277, *P* < 0.057) or its duration (r = 0.265, *P* < 0.072).

### Radiological data of WMHs location

**Fig. 1:**
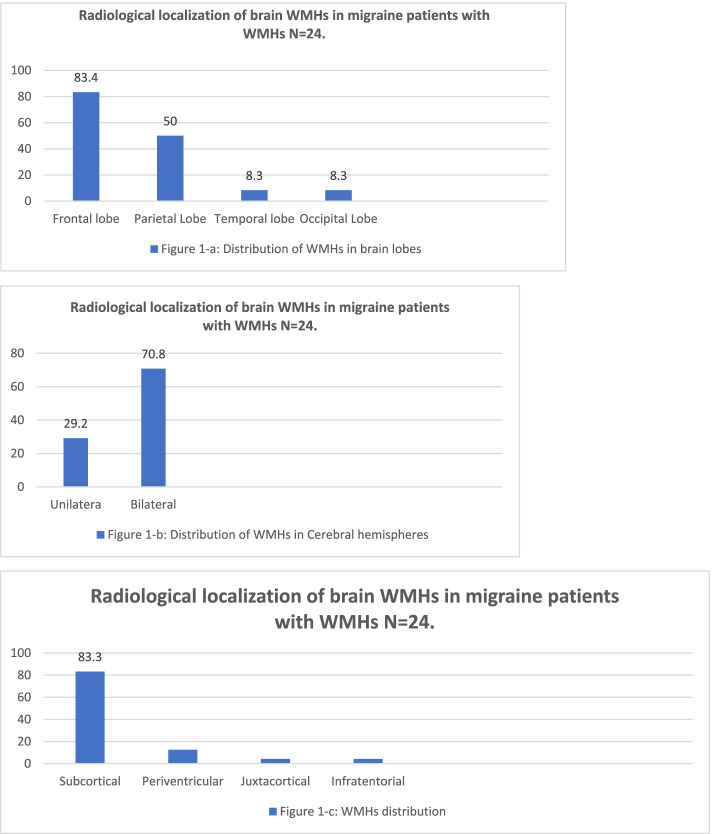
Radiological localization of brain WMHs in migraine patients with WMHs *N* = 24

Figure 1 displays the location of WMHs. WMHs were situated most frequently in frontal lobes (83.4%), less common in parietal (50%) followed with temporal lobes (8.3%), and occipital lobes (8.3%); *P* < 0.018). Most of WMHs were located in both hemispheres (70.8%). WMHs, located mostly subcortically (83.3%), uncommon peri ventricularly (12.5%), infratentorial (4.2%) and (4.2%) at juxtacortical area.

**Fig. 2:**
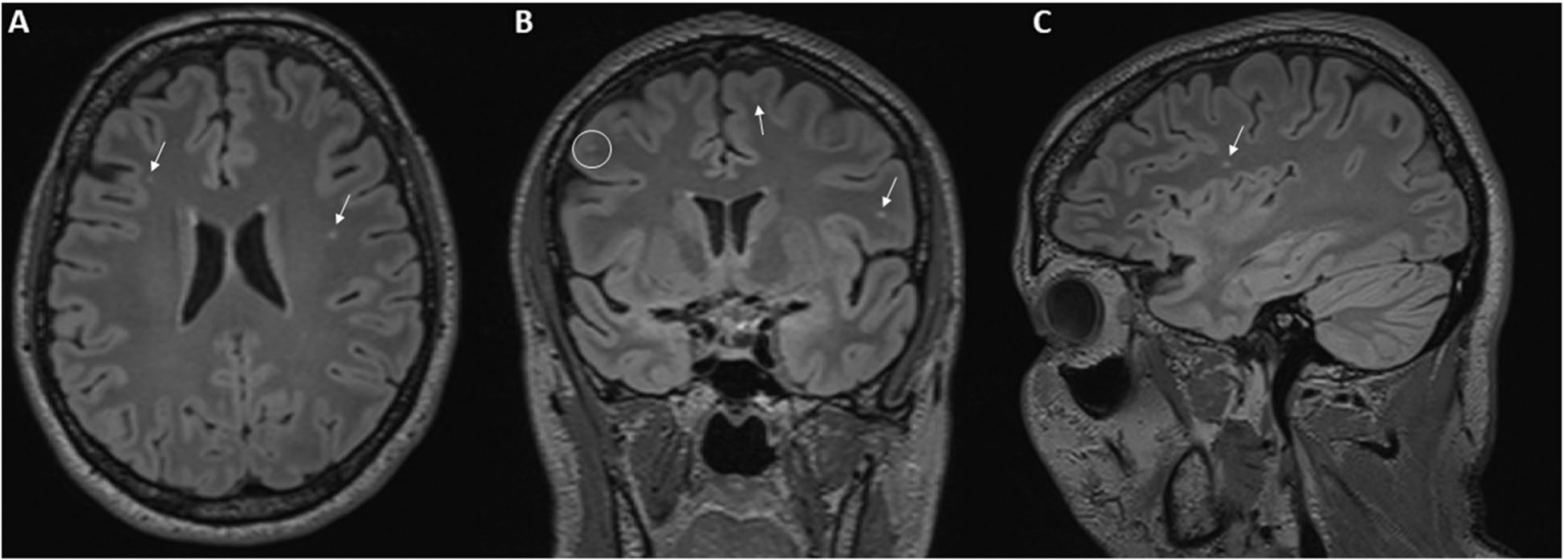
Multiplanar reformats of 3D-FLAIR MRI sequence in a 46 year old lady having migraine over 10 years duration showing different deep white matter hyperintensities (arrows) in axial (**A**), coronal (**B**), and sagittal (**C**) planes. A juxtacortical hyperintensity is also shown abutting the inner margin of the cortex at right middle frontal gyrus (encircled in **B**)

Figure 2 displays MRI image of 46-year-old lady having migraine over 10 years duration. MRI showed different deep white matter hyperintensities.

## Discussion

In this study, we have investigated the frequency of WMHs, their significance, and possible risk factors in migraine patients. We excluded subjects with diseases that can be associated with the presence of brain WMHs without migraine to avoid results biases. The pathophysiological mechanisms of WMHs in migraine are not yet fully understood. It could be a brain injury due to activated metalloproteinases during cortical spreading depolarization [35], ischemic microvascular disturbances with subsequent regional hypoperfusion of the brain [36], micro embolism, hypercoagulability, and endothelial dysfunction [37]. Oxidative stress is present in migraine both in ictal and interictal states, and this may explain the occurrence of WMHs among migraine patients [38].

This study included 60 migraine patients and 20 healthy controls matched in age and gender. We reported that WMHs were more frequent in our cohorts 40% compared to 10% in the control group. The prevalence of WMHs among migraine patients in our cohort is comparable to previous studies that reported prevalence of WMHs among migraine 43.1%, [39],43.2% [40]. Our results are higher than other studies that reported a prevalence of WMHs as 34.8% [41] and 32.2% [12].

Our study showed that migraine patients with WMHs are significantly older than those without WMHs. WMHs are common findings in the general population and can correspond with the normal aging process. Aging is an independent contributor to the prevalence [42]. This study included only subjects < 60 years to avoid any biased results related to the prevalence of WMHs in older age. The mean age of migraine patients with WMHs was 45 years old compared to 35 years of patients without WMHs. Our result is in agreement with Alkhaffa et al. who reported mean age of migraine patients with WMHs was 41 years old compared to 32 years of patients without WMHs [42]. Also, Xie et al. result is in agreement with our result mean age of their cohort WMHs aged 39 years compared to 31 years of those without WMHs [41].

Our study did not report that gender is a risk factor of WMHs in migraine. This result was in agreement with other studies that did not report a significant association between gender and the presence of WMHs [12, 39, 43].

Our study observed the occurrence of WMHs in different migraine subtypes similar to a previous study [44]. WMHs were significantly more frequent among migraine patients with aura compared to those without aura. This result is in agreement with previous studies [39–46]. The relation of WMHs with aura can be explained by fluctuations in cerebral blood flow associated with hyper perfusion or hypoperfusion that is modulated by cortical spreading depression from recurrent aura attacks and affect microvascular hemodynamics leading to ischemic injury [44].

We reported that WMHs were significantly associated with longer migraine duration, higher headache frequency, and elevated plasma concentrations of homocysteine. WMHs were significantly associated with the increasing frequency of migraine attacks. We think that repeated vascular dysfunction causes local ischemia and may be the cause of WMHs. Previous reports showed that increasing headache frequency was associated with higher WMHs prevalence among migraine patients [11–47]. More frequent migraine attacks among patients with WMHs is assumed to be due to recurrent migraine attacks causing prolonged oligemia and may damage the small perforating arteries leading to local hypoperfusion and, as a result, the occurrence of WMHs, which is often interpreted as an independent marker of local brain hypoperfusion [13]. More frequent migraine attacks among patients with WMHs could be explained by the possible pathophysiology of migraine-related WMHs. Both ischemic and inflammatory mechanisms have been included, as there is increased cerebral vulnerability to ischemia in migraine patients, whereas there is also evidence of blood-brain barrier disruption with associated release of proinflammatory substances during migraine attacks. Enhanced susceptibility to spreading depolarization, the electrophysiological event underlying migraine, that could be the mechanism of repetitive episodes of cerebral hypoperfusion and neuroinflammation during headache attacks [48].

The longer migraine duration among patients with WMHs is not surprising if we consider the pathophysiology of migraine [49]. During a migraine attack, several intracranial pathologic processes are detectable, including intracerebral hemodynamic changes, local inflammatory responses, excessive neuronal activation, and excitotoxicity, which may all lead to tissue damage [50]. Longer disease duration increases the possibility of WMHs occurrence.

Our study did not report significant relation between the occurrence of WMH and the severity of migraine pain or longer headache attacks as demonstrated in other studies [12, 14, 45].

WMHs were also significantly associated with migraine disability as measured with MIDAS and HITs scores. This relation could be explained by the significant association of WMHs and the increased frequency of migraine attacks. These scores indicate the burden of the disease and its chronicity; therefore, it is not surprising to have such a correlation.

We did not observe a significant association between WMHs, and cognitive dysfunction similar to the results in the EVA-MRI study [15] and other similar studies [16, 45]. Also, the MoCA score did show significant differences among migraine patients and the normal controls.

Mean serum homocysteine serum level was higher in migraine patients compared to normal control and this result is in agreement with previous studies [18, 22, 23]. We found increased homocysteine serum level in migraine patients with WMHs which is in agreement with a previous study [11, 51]. WMHs represent small vessel damage that has been shown by observational and pathologic studies [52, 53] and this explains the association of high level of serum homocysteine with WMHs in this study. Higher levels of plasma homocysteine among migraine patients with WMHs could be explained by the association of high homocysteine with endothelial damage such as thrombosis. Migraine is known to reduce cerebral blood flow that produces a propagated depolarization wave into the brain cortex, cortical spreading depression [54]. Therefore, high levels of homocysteine may be involved in reducing cerebral blood flow and production of the depolarization wave.

However, in other previous studies [45, 55] no association of WMHs occurrence with serum homocysteine level in migraine was found. Our results are in disagreement with Rościszewska-Żukowska study [56] who reported that homocysteine did not correlate with WMHs. This disagreement could be explained by the different cohort population in our study and their study. Their cohort included only female patients with migraine without aura.

The distribution of the WMHs in the brain in this study showed is more frequent in the frontal lobe and subcortical which is confirmed by previous studies that showed the presence of WMHs in the supratentorial region, mainly in the frontal lobes, and mostly located subcortically [12, 44, 47]. Frequent location of WMHs more in frontal lobes most probably indicates a role of mechanisms that determine the frontal localization of pain during a migraine attack, and the extravasation liquid part of the blood during an attack, in the development of these lesions [44]. Another explanation of observation of WMHs in frontal and parietal lobes is that the lenticulostriate branches of the anterior and middle cerebral arteries are the most affected vascular regions [57].

Based on our findings, WMHs in migraine patients can be classified as subcortical deep white matter lesions. Periventricular juxtacortical and infratentorial white matter lesions were uncommon.

## Conclusions

WMHs are frequent radiological abnormalities that are encountered in headache clinics. Older age, longer disease duration, frequent migraine attacks, presence of aura are the main risk factors for WMHs. There was a significant relationship between high serum homocysteine level and the occurrence of WMH. Migraine severity and duration of headache attacks, do not increase the frequency of WMHs. WHMs are located more subcortically and in the frontal lobes. High MIDAS and HIT6 scores were found to be associated with WMHs among migraine patients. Awareness of high prevalence of WMHs among migraine patients to avoid misdiagnosis of other diseases that can affect the white matter of the brain and common in the same age group and gender like for example multiple sclerosis. Further studies on the pathophysiology of WMH are needed.

### Strength of the study

Our strength is that the neuroradiologists were blinded to the clinical data of patients involved in data collection. Inclusion and exclusion criteria included patients without known risk factors for WMH development to avoid result baize. The use of 3-Tesla MRI for better visualization of smaller lesions.

### Limitation of the study

The cross-sectional study does not allow us to know at what stage the formation of WMHs occurred and what conditions contributed to their occurrence. This study included only patients who visited the hospital which may miss other patients with more or less frequency of WMHs. We did not study the effect of preventive treatment on WMH. Symptomatic and prophylactic treatment may influence the headache duration and intensity that proved a risk factor of WMH in this study because subjects used different medications for different periods. Small sample size because of the strict selection criteria.

## Data Availability

The datasets generated and analyzed during the current study are not publicly available because it includes information on the subjects. The data of this study are available upon request to the corresponding author.
